# Trajectories of Emergency Department Use After Incident Functional Disability

**DOI:** 10.1093/geroni/igab046.452

**Published:** 2021-12-17

**Authors:** Katherine Ornstein, Claire Ankuda

**Affiliations:** Icahn School of Medicine at Mount Sinai, New York, New York, United States

## Abstract

Emergency department (ED) visits for older adults with functional disability may represent unmet needs and are often burdensome to patients and families. While it is known that older adults with functional disability use the ED at high rates, this does not capture the heterogeneity of experience after the onset of disability. Using NHATS, we identified a cohort of older adults with incident disability, or who reported they began to receive help with self-care and/or mobility in the prior year. Using the month that they report first receiving help, we linked to Medicare data to assess quarterly patterns of ED use. We used Group Based Trajectory Modeling to assess the trajectories of ED use after disability. We identified three distinct trajectories of ED use: persistently high, declining, and persistently low. We describe the clinical, household, and sociodemographic characteristics associated with likely membership in each trajectory group.

